# The health and social situation of the mother during pregnancy and global quality of life of the child as an adult. Results from the prospective Copenhagen Perinatal Cohort 1959-1961

**DOI:** 10.1100/tsw.2005.111

**Published:** 2005-12-02

**Authors:** Søren Ventegodt, Trine Flensborg-Madsen, Niels Jørgen Andersen, Joav Merrick

**Affiliations:** ^1^ Nordic School of Holistic Medicine, Teglgårdstræde 4-8, DK-1452 Copenhagen K, Denmark; ^2^ Quality of Life Research Center, Teglgårdstræde 4-8, DK-1452 Copenhagen K, Denmark; ^3^ Quality of Life Research Clinic, Teglgårdstræde 4-8, DK-1452 Copenhagen K, Denmark; ^4^ The Scandinavian Foundation for Holistic Medicine, Sandvika, Norway; ^5^ Norwegian School of Management, Sandvika, Norway; ^6^ National Institute of Child Health and Human Development, Ben Gurion University of the Negev, Beer-Sheva, Israel; ^7^ Center for Multidisciplinary Research in Aging, Ben Gurion University of the Negev, Beer-Sheva, Israel; ^8^ Division of Pediatrics, Faculty of Health Sciences, Ben Gurion University of the Negev, Beer-Sheva, Israel; ^9^ Office of the Medical Director, Division for Mental Retardation, Ministry of Social Affairs, Jerusalem, Israel

**Keywords:** Birth cohort, longitudinal study, maternal health, child health, development, global quality of life, QOL, holistic medicine, SEQOL, Denmark

## Abstract

A prospective cohort study (Copenhagen Perinatal Birth Cohort 1959-61) of 7,222 persons was used in order to explore the association between the social and health situation during pregnancy and the global quality of life (QOL) of the adult child 31-33 years later. Two sets of questionnaires were used with one filled out by physicians during pregnancy and one filled out by the adult children 31-33 years later. The questionnaires included mother's situation during pregnancy and global QOL of the child at follow-up: Well-being, life satisfaction, happiness, fulfilment of needs, experience of lifes temporal and spatial domains, expression of lifes potentials and objective measures. The only indicators to have clear connections with a reduced quality of life were the cases of mother's with syphilis (8.5%), mother's congenital malformations (8.8%), low social group (6.9%) and failing contraception (3.8%). The results obtained repudiate the common notion and hypothesis that the mother's situation during pregnancy is highly important for the quality of life that the child experience as an adult. This suggest that the aspects important for quality of life later on are not found solely in early conditions, but instead more dependent on later attitude towards life of that specific person.

## INTRODUCTION

The concept of quality of life (QOL) has become a topic of discussion both in the field of health and social welfare and in political debate[1-15]. In recent years, the concept of quality of life and living "a good life" has been the subject of a number of philosophical and psychological studies[11-18]. Enhancing the quality of life is more and more considered to be an objective in the treatment and prevention of illness together with the provision of psychosocial support. During the past 14 years, the Quality of Life Research Centre in Copenhagen has investigated more than 11,000 Danes and revealed a series of strong associations between QOL and different factors in our lives[19-25]. Our results are built on the validated SEQOL (Self Evaluation of Quality of Life) questionnaire[26-34]. Among our findings, it became increasingly apparent that illness is closely related to the individual's perception of a good life, which therefore made us explore indicators related to quality of life that might be of broad importance for the prevention and treatment of diseases.

Many studies have been conducted in order to examine the long-term effects of factors that happen to the foetus during pregnancy, because this period is thought to be very vulnerable to the child. The hypothesis of this study was that a connection existed between the mother's situation during pregnancy and the quality of life of the adult child 31-33 years later, which to our knowledge has not been revealed in any other studies. The data was provided from the Copenhagen Perinatal Birth Cohort 1959-1961[35], which has been the subject of a number of studies over time, some of which have explored associations between circumstances during pregnancy and the later effects on the child[36-44]. The present study evaluates the factors related to the mother's situation as indicators for describing associations with the later quality of life of the child.

## METHODS

The connections between quality of life and the various early factors presented in this study were based on two sets of responses. The first set was taken from a questionnaire filled out by the physician who treated and covered the pregnancy, and the second set was the grown-up child's own answers to the SEQOL questionnaire (Self Evaluation of Quality of Life Questionnaire) in 1993[26].

The first set of responses is data from the Copenhagen Perinatal Birth Cohort 1959-1961 that began with the examination of 9,006 mothers and their 8,820 surviving children (from a total of 9,125 children) born at the University Hospital of Copenhagen during the 1959-1961 period. With the help of the CPR (Central Person Register) register we succeeded in tracing 7,222 of the original children from the cohort. To ensure that the people drawn from the register were actually the same persons as the original cohort, names and dates of birth were checked as well as the names and dates of birth for their mothers. When we repeatedly compared these data we found a match between the persons of around 98-99%, and therefore, estimate that the accurateness of the match of the "responses 1959-1961" against the "responses 1993" was better than 98%.

The SEQOL questionnaire measures the global quality of life and is a self-administered questionnaire with items rated on a five-point Likert scale. The questionnaire consisted of 317 items and was based on an "integrative" theory of the quality of life. The integrative QOL theory was created to organize a number of theories on the quality of life into a spectrum that spans the extremes of subjective and objective quality of life. These measures are showed below (sample questions from the questionnaire included):

**Subjective measures**

1. Immediate, self-experienced well being ("How are you feeling?")
2. Life satisfaction ("How satisfying is your life?")
3. Happiness ("How happy are you at present?")

**Existential measures**

4. Fulfilment of needs (e.g., "How well are your social needs fulfilled?")
5. Experience of life's temporal domains (e.g., "How do you feel when you are at home?")
6. Experience of life's spatial domains ("How satisfied are you with [each of five domains: self, partner, family, friends, community]"?)
7. Expression of life's potential [some 30 questions on extent to which they are fulfilled]

**Objective measure**

8.Objective factors [some 80 questions on income, status, work etc.]

Replies to each of the questions that constituted these measures are weighted and scored to yield computable numbers between a minimum of 0 and a maximum of 100[26-34]. These numbers were then taken as representing the quality of life of the respondent, expressed in terms of the eight different ways the quality of life had been measured by the questionnaire. Suitably weighted and scored, replies to the first part of the questionnaire constituted variables, the co-variation from which the quality of life could be calculated. Measuring quality of life has been the subject of many disagreements through time. In our research, the global QOL - in the most broad and all-including sense - was the primary outcome measure (dependent variable). The integrative QOL theory made us include 113 items in the SEQOL questionnaire for the calculation of the global QOL[16]. The rest were control questions and questions giving more information about health status, sexuality, philosophy of life, life style, self-perception, and social circumstances. The principle of SEQOL was that a hierarchy of factorsadd up to an abstract total QOL.

In this study, we had to deal with an essential problem. When the statistical connection between 113 life factors and the global QOL was measured, we often had a contribution to the statistical co-variation from the construction of the global QOL measure. This problem turned out to be of little significance, as even the most strongly “constructed” connections did not count for more that $1/15^{th}$ of the total connection. Still, this gave an error of up to 7% in co-variation. As the large connections in our study showed a co-variation of 20% global QOL, or more, the above-mentioned error introduced by the construction of the global all-including QOL measure was generally negligible. It is important to notice that the way our QOL measure was constructed it does not constitute a measuring problem, and we will almost always find a high correlation when N=5-10,000 between QOL and the many factors constituting the global QOL, or the factors related to them. However, we are not looking at the size of the correlation (the statistical significance), but at the size of the statistical co-variation (QOL difference in %) showing the clinical significance[34].

For validation SEQOL was sent to 2,460 persons aged 18-88 years randomly selected from the Danish Central Person Register together with Nottingham Health profile (NHP), Sickness Impact Factor (SIP), and the test-retest reliability correlation was > 0.8, Cronbach’s alpha was 0.75, correlation (r) to NHP was 0.49, to SIP 0.27 ($P<0.05$). Adjustment for health status made the correlation to SIP stronger among the sick ($r=0.41$). For SEQOL 111 respondents were needed to detect 3% difference in QOL. SEQOL is thus valid as it shows a high level of reliability, sensitivity and consistency.

## RESULTS

The results mentioned in the text below and summarized in Table 1 are chosen among many other findings from the prevailing study; for further details we refer to the original table collections[45-49].

**Mother’s general conditions:** The results showed no, or very small, correlations between common conditions of the mother during pregnancy and the quality of life of the child 31-33 years later. No significant associations were found between global QOL and the mother’s age, height and blood type, and only a small significant association was found in relation to the mother’s weight, whereas those who were born of overweight mothers had a slightly smaller quality of life (1.7% less than average).

**TABLE I. Mother's situation.**
**The connection between global QOL and the mother's situation during pregnancy. Only statistically (p<0.05) and clinically significant factors listed. Difference in global QOL was measured according to the Integrated QOL theory[16] and is measured with the validated SEQOL questionnaire[26].**

| Quality of life and ... | QOL difference % * |
|---|---|
| ..washing machine versus soap (indicator of mother's financial state) | 11.3 |
| ..mother's illness (syphilis) before pregnancy | 8.5 |
| mother's congenital malformations | 8.8 |
| social group | 6.9 |
| failed contraception | 3.8 |
| mother's situation: overview | 3.8 |
| marital status at conception (married versus divorced) | 3.0 |
| venereal diseases | 2.4 |
| mother's abortion attempts | 2.2 |
| mother overweight | 2.1 |
| habituation (living alone with the mother) | 2-4% |

*Difference in percentage between the worst and the best off (single events), or calculated with the method of weight modified linear regression (impact of all events)[34].

**Mother's health:** The mother's health was measured merely in objective terms according to the assessment of the physician. The QOL of the adult child showed an enormous resilience to the health of the mother at the time she gave birth. Illnesses before and during pregnancy and medical treatment of the mother showed almost no correlation with QOL. One noteworthy exception was for children born to mothers who had syphilis (8.5%), congenital malformations (8.8%), or other venereal diseases (2.4%), before pregnancy (see Table 1). If the mother had experienced complications in former pregnancies and deliveries, or had had pre-natal complications, no correlation with quality of life of the child 31-33 years later was found.

**Mother's financial status:** Unfortunately, we have no information about the income per year during pregnancy, but instead we used habitation and the way in which the child's clothes were washed as measures for the financial situation (since this was measured in 1959-1961 it was assumed that the washing machine was a clear indication of the material standard of living and thereby an indication of the economic situation). Only 0.3% of the group used a washing machine and their quality of life were found to be 11.3% higher than average. If the child lived with both parents, the standard of habitation showed no variations in QOL, but if the child lived alone with the mother, QOL was found to be a bit smaller (2-4 %). This suggests that having two parents was a better predictor for a high quality of life later in life than the residence of the child during early childhood.

**Mother’s marital status:** Marital status was weakly connected to QOL of the adult child (3% higher quality of life for those whose mother was married), even though it is known that children of unmarried and divorced mothers were situated significantly below the average as regards quality of life.[49] In the cohort, not much information was found about the father, but the father's age showed a connection with quality of life, since a higher age was associated with a lower QOL in the domain of objective factors (there were too few old fathers in the research to confirm the association in the other dimensions of quality of life). Congenital malformations in the father showed no connection to quality of life and neither did the occurrence of having sexual intercourse, or not, during pregnancy. The fact of being adopted showed no significant association with the later quality of life.

**Mother’s pregnancies and contraception:** The course and number of the mother's former deliveries showed no correlation with the QOL in the adult child. Failed contraception was associated with a quality of life 3.8% below average.

**Mother's social situation and work status:** No significant associations were found between QOL and whether or not the mother worked at the time of the one-year examination; nor the amount of time she worked. The mother’s social group showed a small correlation with QOL, as we found a difference of 6.9% between the highest and the lowest group. No significant associations were found according to the educational level of the main provider.

## DISCUSSION

The results obtained repudiate to a certain degree the common notion, and the hypothesis, that the mother’s situation during pregnancy is important for the QOL of the adult child. The only indicators showed to have clear connections with a reduced quality of life in this long-term study were mother’s with syphilis, failed contraception and low social group.

Syphilis is a venereal disease that is statistically linked to sexual activity with different partners. This could be due to severe psychosocial factors such as prostitution, or it could simply be that the mother had many partners, which may indicate that the woman represented a special group. Therefore, it may not be the syphilis in itself that led to a reduced QOL, but rather some underlying psychosocial factors innate to the child through social inheritance. The association between a lower QOL and failed contraception can be explained by a connection between an unwanted child and limited resources of the mother. In the midst of physical, financial and social misfortune this can also explain the association between QOL and low social group. This is in accordance with another prospective study, which showed that children born to mothers in the lowest socio-economic group were more likely to be impaired according to their mental/emotional health later in childhood[50]. However, we found no significant associations according to the educational level of the main provider. Assuming that highly educated parents generally have highly educated children[50], this is in agreement with other studies that highly educated people do not have a higher quality of life than the lower educated[25,50].

In other studies we have shown that the factors important for the global QOL and for good health derive from good relations with the close as well as the distant world and from the person’s overall view of life[24,25,45-49]. What one possesses in objective terms - money, status, work etc. - does not seem to be important for global QOL and is of little importance to self-assessed health. These results indicate that what is really important is not *what* you have, but *how you see, evaluate and experience what you have.*

The person's level of consciousness and responsible attitude towards life and others seems far more important for the global quality of life and health than the person's social status and material wealth. According to these findings, it is not so strange that we did not find any strong connections between the numerous objective factors of the mother's situation during pregnancy and quality of life of the adult child. The results seemed to indicate that our lives as adults are determined by what we ourselves choose to do with our lives as young people and as adults – and only to a marginal degree determined by factors related to our background. This suggests that we as adults have great freedom to achieve a good life despite our background – despite our outcome due to the natural lottery. These reflections are in agreement with the life mission theory which explains how happiness and QOL come from living your purpose of life in the present time and how you succeed by taking responsibility for your life here and now and by expressing the your talents [51-57].

An important limitation of the research is the quality of data. The 1959-1961 data was possibly sensitive to the physician's opinion, but it is not known how uniform the research and data collection methods were at that time. On the other hand, only one gynaecologist did all the data collected on the mothers, so we presume that this fact guaranties some consistency. However, because of very objective and standard forms of the first set of data, an approximate uniformity is guaranteed. On the other hand, very important limitation of the data collected exists, because of their very objective nature. It would have been valuable to have some subjective life evaluations from the mother as to whether she was satisfied with life, whether she thrived in her relationships and in her work, whether she had some really good friends and whether she was a happy person in general. This would reveal more about how the mother's perceptions of her life had an influence on the quality of life of the adult child.

## CONCLUSIONS

Our results showed only small associations between the mother's situation during pregnancy, and the quality of life of the adult child 31-33 years later. Overall, it was only mothers with syphilis, failed contraception and low social group which showed clear connections with a reduced quality of life in the child as an adult. In accordance with other studies this suggests that the aspects important for quality of life are not found in the personal background, or early experiences. Instead, the quality of life is dependent on the personal level of consciousness and attitude towards life, and it seems as if everyone has the freedom to achieve a good life despite personal background.

## ACKNOWLEDGEMENTS

This study was supported by grants from The 1991 Pharmacy Foundation, as well as by supplementary grants from Goodwill-fonden, the JL-Foundation, E. Danielsen and Wife's Foundation, Emmerick Meyer's Trust, the Frimodt-Heineken Foundation, the Hede Nielsen Family Foundation, Petrus Andersens Fond, Wholesaler C.P. Frederiksens Study Trust, Else & Mogens Wedell-Wedellsborg's Foundation and IMK Almene Fond. The research was approved by the Copenhagen Scientific Ethical Committee under number (KF) V.100.2123/91.

## BIOSKETCHES

**Søren Ventegodt, MD,** is the director of the Nordic School of Holistic Health and Quality of Life Research Center in Copenhagen, Denmark. He is also responsible for a Clinical Research Clinic for Holistic Medicine in Copenhagen and noted as a popular speaker throughout Scandinavia. He has published numerous scientific or popular articles and a number of books on holistic medicine, quality of life and quality of working life. His most important scientific contributions are the comprehensive SEQOL questionnaire, the very short QOL5 questionnaire, the integrated QOL theory, the holistic process theory, the life mission theory, and the ongoing Danish Quality of Life Research Survey, 1991-1994 in cooperation with the University Hospital of Copenhagen and the late professor of pediatrics, Bengt Zachau-Christiansen, MD, PhD. E-Mail: ventegodt@livskvalitet.org. Website: www.livskvalitet.org

**Trine Flensborg-Madsen**, **BSc**, is a research assistant of the Quality of Life Research Center in Copenhagen. She is currently finishing her Master of Public Health (MPH) at the Department of Public Health, University of Copenhagen, Denmark. E-mail: tfm@livskvalitet.org

**Niels Jørgen Andersen, MSc**, professor, Department of Innovation and Economic Organization, Norwegian School of Management. This department conducts research and provides teaching in central topics related to innovation, business development, management of global companies, business history and economic organization. Research activities within the Department are related to four core subjects within the discipline: business history, co-operative organizations, business development and entrepreneurship, and finally studies of industries with a special focus on the electricity industry. He is also the dynamic chairman of the non-profit organisation Stiftelsen Holistisk Medisin Scandinavia that aims to support the scientific development, research, and documentation of complementary and holistic medicine in Scandinavia. E-mail: niels.j.andersen@bi.no. Website: http://www.bi.no/users/fgl93013

**Joav Merrick, MD, DMSc**, is professor of child health and human development affiliated with the Center for Multidisciplinary Research in Aging, Zusman Child Development Center, Division of Pediatrics and Community Health at the Ben Gurion University, Beer-Sheva, Israel, the medical director of the Division for Mental Retardation, Ministry of Social Affairs, Jerusalem, the founder and director of the National Institute of Child Health and Human Development. He has written numerous publications in the field of child health and human development, rehabilitation, intellectual disability, disability, health, welfare, abuse, advocacy, quality of life and prevention and has received the Peter Sabroe Child Award for outstanding work on behalf of Danish Children in 1985 and the International LEGO-Prize ("The Children's Nobel Prize") for an extraordinary contribution towards improvement in child welfare and well-being in 1987. E-Mail: jmerrick@internet-zahav.net. Website: www.nichd-israel.com
